# Microbiota-based PBPK model: new approach methodology for assessing the kinetics of drugs and chemicals

**DOI:** 10.3389/fphar.2026.1840947

**Published:** 2026-09-28

**Authors:** Deepika Deepika, Kanchan Bharti, Judit Biosca Brull, Oscar Sabuz, Yemurai Musengi, Vikas Kumar

**Affiliations:** 1 Department of Chemical Engineering, Institut de Recerca Biomèdica Catalunya Sud (IRBCatSud), Universitat Rovira i Virgili (URV), Tarragona, Spain; 2 Department of Pesticides Safety, German Federal Institute for Risk Assessment (BfR), Berlin, Germany; 3 Department of Biochemistry and Biotechnology, Universitat Rovira i Virgili, Tarragona, Spain; 4 Institut de Recerca Biomèdica Catalunya Sud, Center of Environmental, Food and Toxicological Technology (TECNATOX), Universitat Rovira i Virgili, Reus, Spain

**Keywords:** ADME, gut metabolism, microbial kinetics, microbiota, new approach methodology, PBPK, point of departure

## Abstract

Gut microbiota plays an important role in metabolising environmental chemicals and drugs. It affects the susceptibility of the host to toxicity by modifying metabolism, which can be captured by *in-vitro* assays and translated using *in silico* models. *In-silico* models like physiologically based pharmacokinetic (PBPK) models can predict the concentration-time profile of compounds across different tissues and organs. Despite the recognized importance of gut microbiota in shaping absorption, distribution, metabolism and excretion (ADME) profiles for a wide range of compounds, PBPK models do not include gut microbiota either due to the unavailability of data or to reduce model complexity. This inclusion becomes of utmost importance when metabolism by gut microbiota shape the ADME profile. In this review, we outline the key strategies and considerations for integrating gut microbiota into PBPK models as part of New Approach Methodologies (NAMs), covering the essential equations, parameterization approaches, and *in-vitro*-to-*in-vivo* extrapolation (IVIVE) methods required for model development and validation. We also focus on the limitations of the IVIVE methodologies including population-level variability modelling, and the emerging application of machine learning for microbial parameter inference. We further discuss extended compartmentalization, as well as the integration of genome-scale metabolic (GSM) network models to capture microbial pathway diversity. The review also highlights the broad applications of microbiota-based PBPK models, including personalized medicine, translational modelling, compound-microbiota interactions, and the compound-microbiota-diet axis. Additionally, we examine the current regulatory landscape, identifying existing gaps in PBPK guidelines with respect to microbial metabolism, and propose directions for developing harmonized reporting frameworks. Finally, we discuss future directions including multi-omics integration, bidirectional gut-liver signalling. Together, this review provides a practical roadmap for building microbiota-informed PBPK models that improve kinetic predictions and advance personalized risk assessment.

## Graphical Abstract

**Figure:**
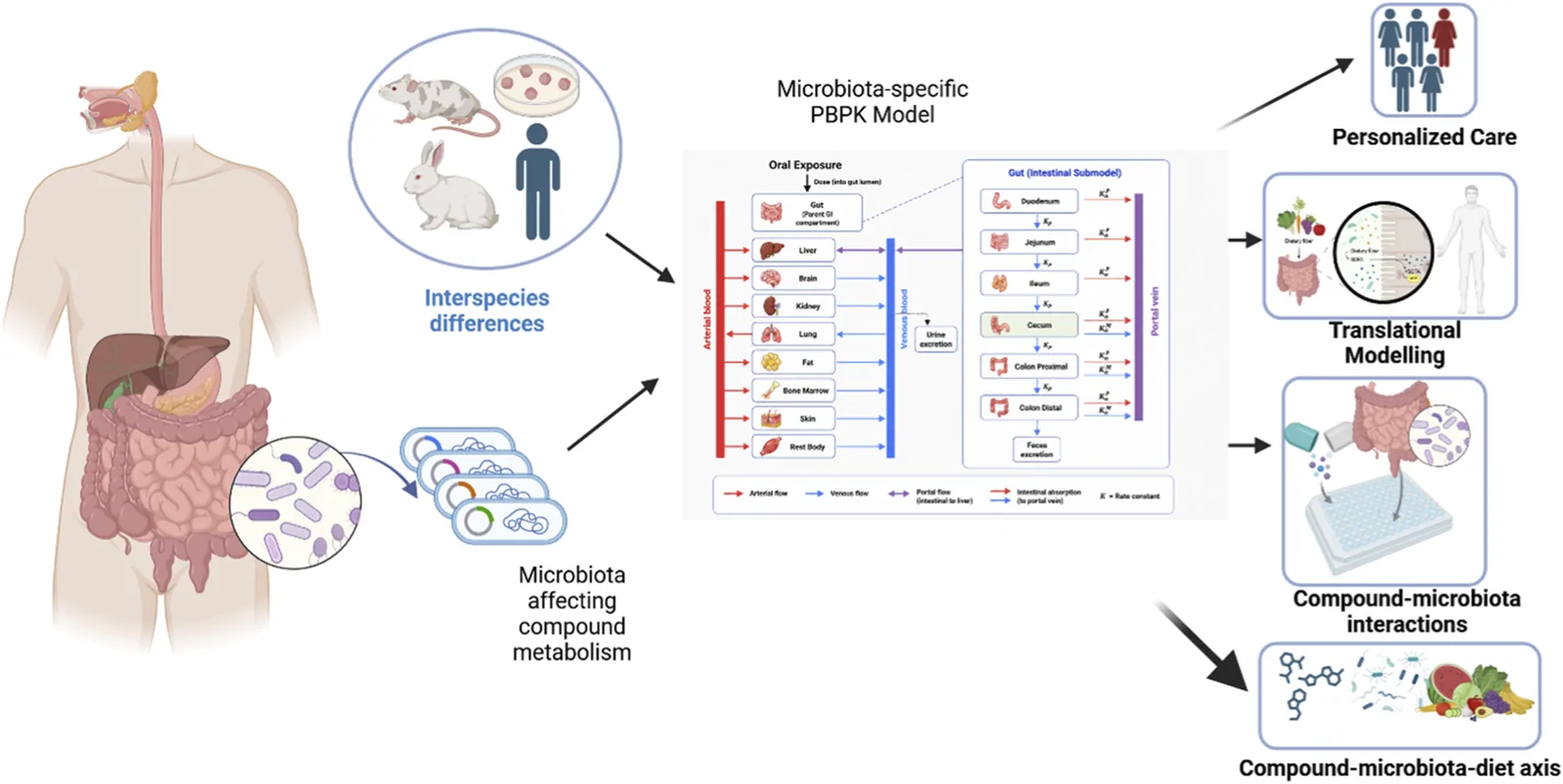

## 1 Introduction

The physiologically based pharmacokinetic (PBPK) models can estimate the absorption, distribution, metabolism and elimination (ADME) of compounds by combining chemical-specific and physiological information. These models form the core quantitative system pharmacology, where ordinary differential equations and compartmental representations of organs are used to describe the pharmacokinetics of chemical substances (Deepika and Kumar 2023). Currently, PBPK models are also used as New Approach Methodologies (NAM), where it is used to predict kinetics, and quantitatively estimate *in-vitro* point of departure (PoD) as a data gap filling technique to translate *in-vitro* toxicity pathway-based findings to assess human health risks (Li et al., 2021). However, microbial metabolism are still not captured in many models, even though its inclusion can substantially improve prediction of interindividual variability in chemical response.

The gastrointestinal tract (GIT) is the primary route of exposure to many xenobiotics through direct or indirect ingestion. Increased consumption of ultraprocessed food products containing preservatives, colorants, emulsifiers, sweeteners and other food processing additives, now accounts for up to half of daily caloric intake in many countries, and has been shown to disrupt the gut microbial environment. This has demonstrated effects on the gut microbial environment, which can act as a precipitating factor for chronic metabolic and neurodegenerative diseases (Balaguer-Trias et al., 2022). Studies already show an increase of 32% in risk of chronic metabolic diseases (Bevilacqua et al., 2024; Martínez Leo and Segura Campos, 2020). The human gut microbiome is now widely acknowledged as a key player in xenobiotic metabolism, capable of altering the bioavailability, biological effects, and toxicity of various chemicals by either activating or deactivating them (Koppel et al., 2017). Such alterations in microbial composition can therefore significantly influence xenobiotic disposition, raising the possibility of inaccurate exposure assessments for environmental chemicals. To achieve accurate and reliable predictions for toxicity, there should be appropriate addressal of complex symbiotic interaction occurring in GIT between host cells and the residing microbiota. Drug-induced gastrointestinal toxicities are among the most common adverse drug reactions during clinical trials (Li et al., 2016), and co-medications or other external factors can exacerbate gastrointestinal side effects. The gut microbial contribution to compound metabolism and toxicity is increasingly recognized but remains an active area of investigation due to the extremely complex relationship between gut microbiota and the host (McCallum and Carolina, 2024). However, there are several established examples of gut microbiota affecting the toxicity of drug at clinically relevant doses. Drug toxicity is significantly modulated through bacterial enzymatic activity. β-glucuronidase-producing bacteria reactivate glucuronidated drugs like irinotecan, regorafenib, and NSAIDs (diclofenac, ketoprofen, indomethacin) in the gut, causing intestinal damage and inflammation; inhibiting this enzyme reduces toxicity and improves drug tolerance. Bacteria also directly generate toxic metabolites, converting brivudine into hepatotoxic bromovinyluracil and reactivating mycophenolate mofetil’s glucuronide form to cause gut inflammation. Additionally, bacterial deconjugation of chloramphenicol produces hematotoxic dehydrochloramphenicol, which re-enters circulation via enterohepatic recirculation, increasing systemic toxicity (Verdegaal and Goodman, 2024).

Gut microbiota plays the most crucial role in maintaining health, where the microflora contributes to food fermentation, pathogen defense, immune system stimulation, and vitamin production (Flint et al., 2012). Multiple studies have identified specific instances of microbiome-mediated drug metabolism, indicating that the impact of gut bacteria on *in-vivo* drug metabolism may be more extensive than previously thought (Koppel et al., 2017). It can be implied that ingested chemicals can affect the gut microbiota, and in turn, the gut microbiota acts on these chemicals, impacting their uptake and metabolism. The wide acknowledgement of the significance of microbiota in health and disease cases which include the gut microbial conversion of the mycotoxin zearalenone into the potent xenoestrogen α-zearalenone, and conversion of hop polyphenols into 8-prenylnaringenin (Possemiers et al., 2005; Possemiers et al., 2006). Other examples include the production of health-promoting urolithins from pomegranate ellagitannins (Tomás‐Barberán et al. 2017), and irinotecan biotransformation (Mahdy et al., 2023). Over 30 drugs (Sousa et al., 2008) and more than 30 environmental chemicals (Claus et al., 2016) have been identified as substrates for intestinal bacteria, affecting the kinetics of compounds with implication on bioavailability and toxicity. This raises the need to understand their disposition in humans by improving the translation modeling from microbial metabolism to clinical pharmacokinetics and chemical pharmacokinetics for risk assessment. Emergence of precision medicines and quantitative systems pharmacology aiming to maximize the efficacy and reduce adverse effects for drugs raises the need to improve computational models like PBPK for capturing ADME profiles and understand the interplay between drug-microbiota interactions at cellular level.

Recent publications have improved our understanding of the gut microbiota as one of the factors determining the pharmacokinetics of drugs and chemicals. Zimmermann-Kogadeeva et al. (2020) developed a mouse PBPK model with an intestinal compartment that includes microbiome drug-metabolizing activity. They also assessed how microbiota influence concentration–time profiles under altered physiology and microbial properties, thereby evaluating the impact of microbiota on drug pharmacokinetics. This model framework could be applied to investigate the microbiome’s influence on multiple drugs and chemicals and may provide a useful starting point for regulators considering this aspect. Stevanoska et al. (2024) emphasized the importance of incorporating gut microbial biotransformation of xenobiotics, with particular focus on approaches used to measure metabolic constants and to scale these kinetic parameters. The authors also highlighted several challenges, including the need for method standardization and the limitations associated with the dynamic nature of microbial metabolic activity. While these papers address some of the fundamental aspects needed for building microbiota-specific PBPK models, our review is to provide an overall framework needed for developing such models and also pointing towards regulatory gaps which exists in PBPK modeling.

It serves as a practical guide for developing robust microbiota-specific PBPK models for both experienced and new researchers. In particular, it presents an equation-driven six-step NAM framework (Section 2.7) with compartment-specific mass-balance equations, microbial intrinsic clearance scaling, and a tiered model-selection guide, moving beyond conceptual discussion to offer practical guidance for model development. It also systematically addresses IVIVE limitations specific to microbial parameters, including fecal fraction scaling, Michaelis–Menten constant assumptions, and interindividual variability. Finally, it evaluates the current regulatory landscape across USFDA, EMA, and OECD frameworks and identifies specific gaps in guidance for microbiota-informed PBPK model qualification, a perspective that has not been examined in depth elsewhere. Together, these contributions position the review as a practical methodological resource for researchers and regulatory scientists seeking to incorporate gut microbial metabolism into PBPK modelling workflows.

## 2 New approach methodologies for microbiome-related kinetics

New approach methodologies (NAMs) are faster and less expensive alternatives for generating new information for risk assessment. According to EFSA, it is the first choice for data generation on newer chemicals which can be used as a part of the Integrated Approaches to Testing and Assessment (IATA) for chemical risk assessment. As stated by USFDA, the high amount of time, cost and limitations of the number of chemicals to be tested by the traditional studies involving rodent or any other animal, the development of reliable and valid NAMs are the need of the hour (USFDA, 2024; Usmani et al., 2024).

Traditional microbiota research is dominated by studies based on animals (Cani et al., 2008) or on human biomonitoring data (Wang et al., 2021; Yamaguchi et al., 2016). Within the NAMs space, numerous *in-vitro* studies which include static and continuous batch fermentation systems have been conducted using intestinal cell lines and complex microbial consortia, enabling characterization of the microbial community function in the gut (Brodkorb et al., 2019). *In-vitro* studies conducted on gut microbiota have also led to the emergence of “culturomics,” which combines diverse culture media and optimized growth conditions with 16S rDNA sequencing and mass spectrometry to enable the identification and isolation of fastidious bacterial species from the human gut. These studies are important because they help improve treatments for diseases linked to gut microbiota imbalance, such as through fecal microbiota transplantation (Yousi et al., 2019). There are NAMs such as colonic fermentation models which tend to decrease the interindividual variability in finding out the factors responsible for influencing the composition and metabolic capacity of gut microbiota. There is one such type of continuous fermentation model for generating more reliable kinetic data is the continuous multi-reactor system PolyFermS, where one fecal-inoculated reactor feeds several parallel reactors under matched physiological conditions. In this setting, fecal microbiota of healthy elderly patients was used to characterize microbial diversity (Fehlbaum et al., 2015). Applying this system to xenobiotics, by testing a range of compound concentrations on the same microbiota, would allow more reliable estimation of microbiota-relevant kinetic data than single-batch incubation.

While *in-vitro* studies have generated many important findings to answer the how and why of microbiota functionalities, *in silico* investigations have only just begun to scratch the surface. PBPK modeling is one such *in silico* approach, enabling the quantification of metabolism by both the host and the microbiota (Zimmermann-Kogadeeva et al., 2020), thereby facilitating a more accurate representation of human health metrics. In the following subsections, we have discussed modifications to PBPK modeling that incorporate metabolic contributions from both the gut microbiota and the host, along with examples of successfully developed models.

### 2.1 Microbial metabolism and effect on compound kinetics

Gut microbiota plays a critical role in the biotransformation of xenobiotics, including drugs, food-derived compounds, and toxins, influencing their bioavailability. Therefore, it is relevant to include the microbiota in PBPK models as it can helps us to better predict the efficacy and toxicity of these compounds and allows the prediction of the interindividual variability in their metabolism.

Depending on the compound, gut microbial metabolism can vary throughout the GIT but most dominating regions are small intestine (duodenum, jejunum and ileum) and large intestine (cecum, colon and rectum). Microbial density also varies throughout the GIT with 10^3^–10^4^ CFU/mL in duodenum, 10^7^–10^8^ CFU/mL in ileum, 10^10^–10^11^ CFU/mL in cecum and proximal colon and 10^12^ CFU/mL in distal colon and rectum which plays an important role in shaping metabolism. There are predominant reactions which happens during metabolism like hydrolysis, reduction, sulfation, inactivation, deconjugation, deacetylation, functional group transfer reaction, etc., leading to toxic or non-toxic metabolites (Maciel-Fiuza et al., 2023). Important point worth mentioning here is that some of these metabolites play an important role in enterohepatic recirculation (EHR) especially extending the half-lives and increasing the concentration in plasma sometime leading to improved efficacy of drugs while in some cases leading to increased toxicity (Stevanoska et al., 2024).

For integrating the metabolic aspect in PBPK models, crucial point is understanding of human gut microbiota, microbial kinetics and translation of data for human health risk assessment. In last few years, with the use of next-generation sequence technology and metabolomics, there has been increased understanding about microbial composition and their role in metabolism. In general, GI tract of healthy adult consists of two dominant bacterial phyla, Firmicutes (gram positive) and Bacteroidetes (gram negative) along with lower abundance of Proteobacteria, Actinobacteria and Verrucomicrobia (Ghosh and Pramanik, 2021). There are multiple evidences about how drugs and chemicals are being metabolised with specific reactions and mechanisms through microbial modifications. This knowledge can be explored for developing microbiota-specific models. For example, orally administered neoprontosil and prontosil undergoes azoreduction by gut bacteria to sulfanilamide which is having antibacterial effects (Kim, 2015). Another example is sulfasalazine used to treat inflammatory bowel disease (IBD) is getting metabolised in colon forming two metabolites through reduction of azo bond by bacterial azoreductase. One of metabolite called 5-aminosalicyclic acid (5-ASA) shows anti-inflammatory activity. There are many of such examples of xenobiotics getting metabolised into active or inactive forms by gut microbiota which represents interesting scenarios for modelling this and evaluating effect on kinetics especially plasma and other organ concentrations with time (Table 1).

**TABLE 1:** Examples of drugs and chemicals metabolized through gut microbiota.

Drug/chemical	Use	Metabolism	Effect on ADME	Bacterial species involved	References
Zearalenone	Mycotoxin exerting estrogenic effect	Reduction transformation of ZEN to α-ZEL and β-ZEL	Altered bioavailability, increased estrogenicity via α-ZEL, modified metabolism and excretion pathways	Not mentioned	Mendez-Catala et al. (2020)
Daidzein	Food borne compound exerting estrogenic effect	Daidzein is converted into dihydrodaidzein which ultimately converts into S-equol	S-equol a biotransformation product of diadzein is primarily responsible for the estrogenicity	Anaerobic bacterial strain	Wang Q. et al. (2022)
*S*-verapamil	Calcium-channel blocker (therapeutic)	Lowered liver metabolism due to altered microbiota in Corhn’s disease	8.7-fold increase in concentration and 9.6-fold higher Cmax in active diseased patients w.r.t healthy volunteers.	Not mentioned	Alrubia et al. (2022)
Midazolam	Sedation (therapeutic)	Lowered liver metabolism due to altered microbiota in Corhn’s disease	Concentration of midazolam increased 5.3-fold in active diseased patient w.r.t healthy patients.	Not mentioned	Alrubia et al. (2022)
Tobramycin	Antibiotic (therapeutic)	Intestinal deconjugation and Intestinal bile acid reuptake	Reduction in residual plasma *C*_max_ of deoxycholic acid, from 41.5% to 24.2%. Conjugated bile acid concentrations also decreased reflecting enterohepatic circulation of bile acids.	*Clostridium, Bacteroides, Lactobacillus,* and *Enterococcus*	Zhang et al. (2025)
Perfluorooctane sulfonate (PFOS)	Environmental chemical used for many applications like protective coating material	Affects lipid metabolism, oxidative stress, inflammation, TCA cycle, glucose, and amino acid metabolism	Host metabolome is affected due to alteration in gut microbiome as a result of PFOS exposure	*Firmicutes* and *Bacteroidetes*	Zhang et al. (2020)
Ginsenosides	An active compound of ginseng proven to ameliorate obesity	Affects the short chain fatty acids, absorption of lipopolysaccharides, changes the intestinal mucosal permeability and hence improving the inflammation reaction affecting the obesity	Non polar secondary metabolites having stronger pharmacological effect are formed by the action of gut microbiome	^#^ *Bacteroidetes, Proteobacteria, Bifidobacterium, Anaerostipes*	Zhao et al. (2023), Zhuang et al. (2021)

^#^ Strain affected in humans is reported.

Metabolism by gut microbiota not only affect drugs but also environmental chemicals altering their mechanisms or such chemicals sometimes cause dysbiosis of gut microbiota. Endocrine disrupting chemicals like bisphenol A (BPA) is being metabolized into BPA-glucuronide (BPAG) in liver through UGT (UDP-glucuronosyltransferase) enzymes (Deepika et al., 2022) and BPAG is excreted into small intestine through bile which is further deconjugated back to free BPA by β-glucuronidase in cecum of rat (Sakamoto et al., 2002). This leads to increased systemic half-life of BPA by EHR of metabolite and parent chemical with involvement of liver enzymes and gut microbiota (Liu et al., 2022). Such kind of finding along with quantitative data can be fed into PBPK models to simulate longer half-lives and kinetics during repeated exposure helping in associating it with increased adverse effects. Another study published on BPA and its analogs (BPS, BPF, and TMBPF) showed that all bisphenols reduced the abundance of Bacteroidetes phylum with some bisphenols reducing short chain fatty-acids production while others affecting caco-2 cell viability (Emanowicz et al., 2024). Further, BPA-induced dysbiosis has been shown to alter the production of key microbial-derived compounds, including short-chain fatty acids (SCFAs), aromatic amino acid derivatives, and pro-inflammatory compounds such as lipopolysaccharide (LPS). These shifts in microbial metabolism are associated with impaired intestinal barrier integrity, systemic inflammation, and metabolic disturbances, ultimately contributing to the development of obesity, insulin resistance, and immune imbalances (Gálvez-Ontiveros et al., 2020). Such kind of studies open up the space for evaluating environmental chemicals and drug interaction considering into account variations in microbial community which in turn can increase or decrease drug bioavailability and efficacy.

### 2.2 Integrating microbiota into PBPK models

Incorporating the gut microbiome into PBPK models enables clear differentiation between metabolites produced by host tissues and those generated by gut microbes, especially when both contribute to the formation of the same metabolite (Zimmermann-Kogadeeva et al., 2020). For integrating microbiota in PBPK models, data generated using experimental method is of utmost importance. With the advent of NAMs, a lot of data is being generated using *in-vitro* models to reduce uncertainty and animal usage. The data generated using *in-vitro* models are further extrapolated to simulate the *in-vivo* conditions where the role of IVIVE comes into play.

The IVIVE of gut microbial kinetics is the translational process of scaling kinetic data derived from lab assays for the prediction of lab *in-vivo* microbial metabolism. This is a bottom-up approach which develops the understanding of the effect of microbiome on drugs or chemicals. In the current approaches the fecal slurry of animals or human are being utilized to isolate and incubate microbiota under anaerobic conditions as the composition in fecal samples is representative of colon microbial community. Aliquots of fecal slurry are being prepared based on species from which samples are collected and stored at −80 °C for further use. The velocity (Vmax) of metabolite formation is derived using the ratio of the geometric mean bacterial count in the colon to the mean bacterial count in the stool samples. Since Clint is a definite value, the ratio of Vmax/Km must be maintained which is achieved by scaling Vmax accordingly. To simulate the *in-vivo* condition, inter-individual variability in luminal metabolism is accounted for using the coefficient of variation of the colon-to-fecal bacterial count ratio as %CV of metabolic activity in PBPK simulations (Cheng et al., 2026; Sender et al., 2016). Similar approach has been used recently for determining the microbial metabolism of prodrugs where metabolism converts them into their active form. There also, for doing the IVIVE, the compound is incubated with simulated colonic bacteria. Later the degradation half-life is used for estimating the Clint, which is used in the PBPK model as an input parameter (Kostantini et al., 2022). These approaches are critical for regulatory decision-making and must be transparently communicated when reporting microbial IVIVE results.

*In-vitro* incubation is being carried out with different concentration of fecal slurry (1.4%–20%) and are being exposed to compound concentration over time under anaerobic conditions. These are being incubated overtime and sampled depending on the compound properties. For instance, to evaluate metabolism for zearalenone (ZEN), linearity across fecal slurry concentrations was established using a fixed 5-h incubation, while linearity over time was established separately using a fixed 5% fecal slurry sampled hourly for a total of 8 h. Based on these optimization experiments, the final kinetic incubations were carried out using 5% fecal slurry incubated for 5 h. Samples were centrifuged and the supernatant was kept for LC-MS/MS analysis, using fecal samples from rats, pigs and humans. Whenever performing experiments like this, incubation with human liver microsomes and S9 fractions is also required to evaluate hepatic metabolism along with GIT metabolism. Timing of incubation can vary based on compound but for ZEN, it was performed for 10 min for microsomes and 5 min for S9 (Mendez-Catala et al., 2020). Samples can be centrifuged and used for quantitative analysis.

*In-vitro* data obtained through experiments explained in above sections needs to be evaluated and based on the data trend linear-conversion can be applied. If metabolism follows Michaelis-Menten kinetics then consideration of the factors like apparent maximum velocity (Vmax), the constant (Km) for the metabolism due to liver and gut microbiota in the Michaelis-Menten equation (Equation 1) along with catalytic efficiency (kcat) can be determined. After this step, comes the conversion of IVIVE which can be further integrated to calculate intrinsic clearance using first-order reaction kinetics or Michaelis-Menten kinetics. Vmax obtained through incubation of fecal samples in pmol min^−1^ g^−1^ feces can be scaled to whole body by including body weight fecal fraction which is 0.0164 for rats (4.1 g of defecation volume/day) and 0.0018 for humans (128 g defecation volume/day) (Mendez-Catala et al., 2020). Subsequently for rat liver S9 and human liver S9, scaling is done by assuming 143 mg S9 protein g^−1^ (Wang Q. et al., 2022) and 120.7 mg S9 protein g^−1^ for rat and human (Ren et al., 2024). For intestinal Vmax, values are 37.1 and 35.2 mg S9 protein g^−1^ to whole tissue for rat and human. A typical Michaelis-Menten equation explaining the enzyme saturation during metabolism of compounds has been shown below (Equation 1).

$$V=\frac{V_{\max}*\left[S\right]}{K_{m}+\left[S\right]}$$ (1)

Beyond the Michaelis-Menten equation for microbial enzyme kinetics (Equation 1), a complete mechanistic description of gut microbial contributions to PBPK models requires equations capturing intestinal wall absorption, microbial intrinsic clearance, and their interplay with hepatic clearance. Intestinal wall absorption from each GIT compartment into the portal circulation is driven by passive permeability and is calculated from the *in-vivo* apparent permeability coefficient (Papp,*in-vivo*), estimated from Caco-2 cell data using established *in-vitro in-vivo* correlations (Mendez-Catala et al., 2021; Sun et al., 2002). The absorption rate from compartment i (μmol h^−1^) is provided as Equation 2.

$$Absorption\;rate_{i}=Papp,in\;vivo*SA_{i}*C_{lumen,i}$$ (2)

SA_i_ = luminal surface area of compartment i (cm^2^); C_lumen,i_ = luminal concentration (mM). Surface areas differ across GIT segments and species: rat small intestine 94 cm^2^, human small intestine 7,200 cm^2^ (72 dm^2^), human large intestine 4,700 cm^2^ (47 dm^2^) (Mendez-Catala et al., 2021). For compounds undergoing active intestinal transport (e.g., bile acids via the ASBT transporter), absorption is additionally governed by a Michaelis-Menten transport term provided as Equation 3 (Zhang et al., 2025).

$$v_{transport}=\left(Vmax,ASBT*\;\left[S\right]\right)/\left(Km,ASBT+\left[S\right]\right)$$ (3)

Vmax, ASBT = maximum transport velocity (μmol h^−1^); Km, ASBT = Michaelis constant (μmol L^−1^); [S] = substrate concentration.

The microbial intrinsic clearance (CL_int,mic_) in each gut compartment is derived from *in-vitro* Michaelis-Menten kinetic parameters scaled to whole-body conditions:

$$C_{L\mathrm{int},mic}=\left(V_{\max,mic}\times \;60/Km\right)\times f_{feces}\times BW\times 1000$$ (4)

Vmax,mic = *in-vitro* microbial Vmax (pmol min^−1^ g^−1^ feces); 60 = conversion factor from minutes to hours; Km = Michaelis constant (μM); f_feces = species-specific fecal fraction of body weight (rat: 0.0164; human: 0.0018); BW = body weight (kg); 1,000 = conversion factor from kg to g, required because f_feces is defined on a gram body weight basis.

This compartment-specific microbial CLint, derived entirely from fecal incubation data (a NAM input), can then be incorporated into the gut mass balance alongside hepatic intrinsic clearance to describe their combined effect on systemic bioavailability.

In this section, important aspects about microbial IVIVE integration into PBPK models have been discussed. However, it is important to highlight a few experimental gaps of these approaches. The fecal slurries are used as a practical surrogate for the microbial community, and while the colon hosts approximately 70% of intestinal bacteria and fecal communities are broadly comparable to colonic communities, they do not capture the distinct microbial communities of the small intestine (Mendez-Catala et al., 2021). For compounds where intestinal metabolism is relevant, fecal-based IVIVE may underestimate metabolic contributions from upper GI segments. Besides this, the Km values derived from *in-vitro* incubations are conventionally applied without further extrapolation to the *in-vivo* situation. This approach does not account for the fact that *in-vivo* conditions which ranges from pH gradients across the GIT, competition from co-ingested substrates, mucus viscosity, and varying transit times, may alter effective enzyme affinity in ways that cannot be captured by a single *in-vitro* Km value. Also, the fecal mass scaling factors used in IVIVE are population-level averages that do not reflect interindividual variability arising from age, sex, diet, disease state (e.g., inflammatory bowel disease, dysbiosis), or antibiotic use, all of which can significantly alter microbial biomass and enzymatic activity. These variabilities are not currently propagated in most published microbiota PBPK models, leading to predictions that represent a mean microbial phenotype rather than the full distribution of expected exposures in human population.

### 2.3 PBPK model development strategies with microbiota

Different type of PBPK models exists varying from one-compartment to multi-compartmental models depending on the data-availability and aim of the model. In general, model combines the prior knowledge of physiology and chemical-specific information to simulate compound concentration-time profile. A whole-body PBPK model typically contains lung, liver, brain, stomach, spleen, muscle, kidney, gonads, adipose tissue, thymus, skin and pancreas linked by arterial and venous blood compartments through blood flow and partition coefficient (Figure 1) (Deepika and Kumar 2023). While there have been few success stories about PBPK models, there are still some aspects which needs to be addressed to maximize their utility for predicting kinetics of drugs and environmental chemicals. One such aspect is modelling for absorption and metabolism in gastrointestinal tract (GIT) considering microbiota including physicochemical properties and interplay with existing physiological process in GIT.

**FIGURE 1:**
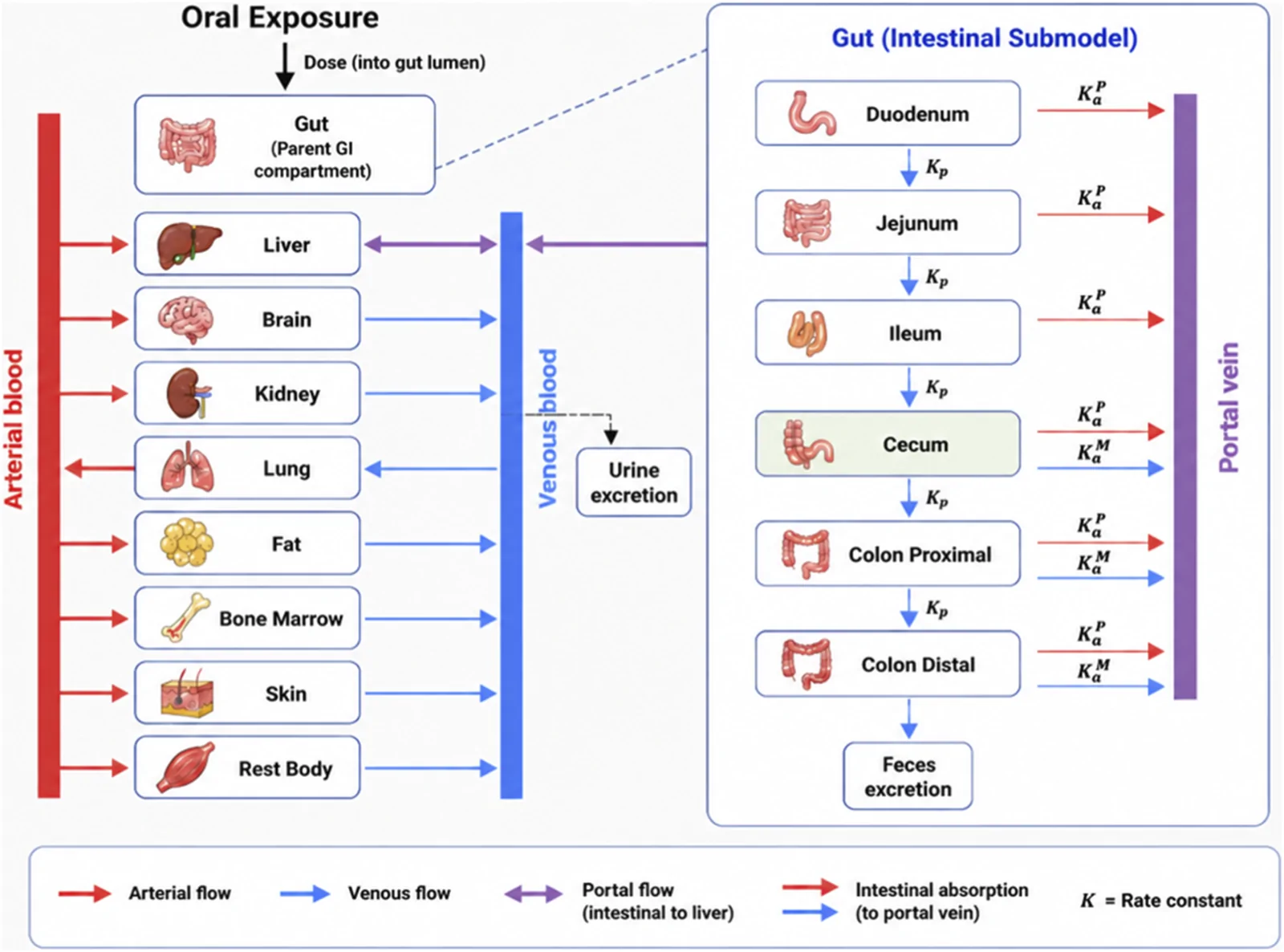
Schematic representation of microbiota-PBPK model with 13 main compartments and seven sub-compartments of gut (intestinal submodel). Green coloured compartment represents the site where major microbial metabolism is happening. $K_{a}^{P}$ and $K_{a}^{M}$ means absorption rate constant of parent and metabolite respectively, showing the action of microbiota on the parent drug/chemical (P) to transform it in metabolite (M).

In general, we keep GIT as one compartment in the PBPK model and try to make it simplistic but with growth of knowledge about physiology and high throughput *in-vitro* data, it is possible to describe movement of compounds from one region in GIT to next with estimations from permeability, dissolution experiments and metabolism by microbiota in large intestine (Enright et al., 2017) Microbiota can be either considered as metabolic compartment, or metabolic parameters can be incorporated in different compartments of GIT in PBPK model (Stevanoska et al., 2024). There is an example where PBPK model integrating microbial deconjugation (sourced from fecal incubations) and ASBT-mediated bile acid reuptake (sourced from Caco-2 Transwell assays) are used to predict tobramycin’s impact on systemic bile acid levels (Zhang et al., 2025). Consideration of discreet compartments of stomach, small intestine tissue and lumen and large intestine lumen instead of a single gut compartment also becomes important for improved predictions (Mendez-Catala et al., 2021). This facilitates the formation of primary metabolite which further gets metabolized in liver.

Currently, we have some commercial softwares from GastroPlus, Certara Simcyp, and MATLAB which account for specific processes for GIT including release of drugs from dosage forms, decomposition, first-pass metabolism, efflux and gut microbial metabolism. But most of these success stories are from commercial software with limited knowledge and information available on how to model microbiota and its integration in generic PBPK model.

To fully capture the dynamic movement of compounds through the GIT and their interaction with regionally distributed microbiota, the mass balance for each discrete intestinal lumen compartment “i” must simultaneously account for transit from the preceding compartment, absorption into the intestinal wall, and microbial biotransformation. The general differential equation governing the amount of parent compound (A_P,i) in compartment i is:

$$\frac{dA_{P,i}}{dt}=k_{t}*A_{p,\left(i-1\right)\;}-k_{t}*A_{P,i}-\left(P_{app,in\;‐vivo}*\frac{SA_{i}}{1000\times V_{i}}\right)*A_{P,i}-\left({CL}_{\mathrm{int},mic,i}/V_{i}\right)\;*\;A_{P,i}$$ (5)

k_t_ = first-order intestinal transit rate constant (h^−1^) = 1/t_transit_; V_i_ = luminal volume of compartment i (L); 1,000 = conversion factor from L to cm^3^; CL_int,mic,i_ = microbial intrinsic clearance in compartment i (L h^−1^) from Equation 4. The fourth term is the microbiota-specific addition that distinguishes this framework from a standard PBPK gut compartment.

Gastric emptying into the first intestinal compartment follows first-order kinetics:

$$\frac{dA_{stomach}}{dt}=-k_{sto}*A_{stomach}$$ (6)

*k_sto_* = stomach emptying rate constant (*h^−1^*); half-life ≈15 min in both rat and human (Mendez-Catala et al., 2021). The small intestinal lumen is subdivided into multiple sub-compartments (e.g., seven in the ZEN PBK model) to adequately describe regional transit and absorption dynamics.

Equations 7–9 represent an alternative, simplified steady-state formulation of oral bioavailability based on the classical well-stirred extraction-ratio model, rather than a component to be solved simultaneously with the dynamic mass-balance equations (Equations 5 and 6). While Equations 5 and 6 provide a fully mechanistic, time-resolved description of compound transit, absorption, and microbial biotransformation across discrete GIT sub-compartments, Equations 7–9 offer a lower-resolution, algebraic approximation of the same competitive first-pass extraction process, useful when only a steady-state estimate of oral bioavailability is needed rather than a full concentration-time profile. Because CL_int,mic_ and CL_int,hep_ are common to both formulations, the two should not be combined additively; Equations 7–9 instead serve as a simplified, minimal-PBPK-tier alternative to Equations 5 and 6, consistent with the model hierarchy described in Section 2.3. The interplay between intestinal microbial clearance and hepatic intrinsic clearance in determining oral bioavailability is expressed through the well-stirred model. The overall oral bioavailability (F_oral_) is the product of the fraction absorbed (F_a_), gut availability (F_g_), and hepatic availability (F_h_):

$$F_{oral}=F_{a}*F_{g}*F_{h}$$ (7)

$$F_{g}=Qgut\;/\;\left(Qgut+fu*\;{CL}_{\mathrm{int},mic}\right)$$ (8)

$$F_{h}=\frac{Q_{h}}{\left(Q_{h}+Fu*{CL}_{\mathrm{int},hep}\right)}$$ (9)

Q_gut, Q_h = gut and hepatic blood flow (L h^−1^); f_u = unbound fraction in blood; CL_int,mic_ = total intestinal microbial intrinsic clearance (from Equation 4); CL_int,hep_ = hepatic intrinsic clearance.

Equations 7–9 explicitly capture the competitive relationship between microbial and hepatic first-pass extraction: compounds with high CL_int,mic_ show reduced F_g_, lowering systemic exposure independently of hepatic metabolism. For compounds undergoing enterohepatic recirculation (EHR), microbial deconjugation regenerates the parent compound from biliary conjugates, effectively reducing net first-pass extraction and extending systemic half-life an effect that requires explicit representation of the biliary input into the gut lumen and the subsequent microbial deconjugation and reabsorption, rather than being captured implicitly through CL_int,mic_ alone. The individual steps of HER, biliary secretion, microbial deconjugation, and reabsorption are rarely identifiable as independent parameters from standard pharmacokinetic data. Published PBPK models of compounds undergoing EHR, such as the human bisphenol A model of Yang et al., 2015, therefore represent the overall recirculation process using a single lumped first-order term rather than resolving each mechanistic step individually. The present framework follows this same convention, treating EHR as a composite contribution within CL_int,mic_ rather than as a separately parameterized multi-step process (Yang et al., 2015).

In a PBPK model, mass balance is applied to all compartment, where the rate of change of compound amount in the tissue equals what the arterial blood brings in minus what the venous blood carries out, plus or minus any local source or sink. Mass balance is the essence of PBPK model and should be checked before starting the model simulations.

#### 2.3.1 Extending compartmentalization with gut microbiota contributions

As discussed earlier that the consideration of discreet compartments instead of one single gut compartment can be helpful in predicting the chemical exposure. One such approach which is widely accepted in pharmaceutical industry is Advanced Compartmental Absorption and Transit (ACAT) model that further help in extending the understanding of absorption of drugs accounting the presence of microbiota in each compartment. The application of ACAT model for the gut absorption prediction of drug is widely explored while this is at a very nascent stage for the prediction of environmental chemicals. The absorption of environmental chemicals is usually considered to be 100%. The integration of gut microbiota in the ACAT model can simplify the prediction since the presence of different strains of microbiota in different gut segments and their respective effects on metabolism can be estimated.

However, a model called PECAT (Probabilistic Environmental Compartmental Absorption and Transit) has been developed by Lin and Chiu by calibrating the permeability datasets obtained from different sources (*in-vivo*, *ex-vivo* and *in-vitro*) on drug permeability and fractional absorption, while accounting for critical aspects like the difference between permeability measured in Caco-2 cells and that observed in the human jejunum, and the variation in *in-vivo* permeability across different regions of the gastrointestinal tract (Lin, 2023). This extends the ACAT modelling framework to environmental chemicals with explicit, quantified uncertainty characterization. Unlike the proprietary ACAT model confined to GastroPlus, PECAT is implemented in open-source platforms (GNU MCSim in R) and can be programmed in any language, allowing greater transparency, reproducibility, and flexibility. In the PECAT model, the parameters were calibrated using permeability and fractional-absorption data from 70 drugs and nine environmental chemicals, accounting for the discrepancy between Caco-2 apparent permeability and *in-vivo* jejunal permeability, and for segment-specific permeability variation across the gastrointestinal tract. Using Monte Carlo simulation to measure these uncertainties, the best-performing model configuration achieved a mean absolute error of 13% and 14% for drugs and environmental chemicals. The model predicts the fraction absorbed of 78% for environmental chemicals and 81% for drugs within ±20% of the observed values with independent validation on a supplementary dataset (162 data points) confirming similar performance. The probabilistic nature of PECAT provides confidence intervals for fraction absorbed and systemic concentrations, making it particularly advantageous for uncertainty characterization in chemical risk assessment (Lin, 2023). A comparison of both the models has been represented in Table 2.

**TABLE 2:** Comparison of ACAT and PECAT models for oral absorption prediction.

Feature	ACAT model	PECAT model
Origin	Developed for pharmaceuticals.	Adapted for environmental chemicals.
Implementation	Proprietary model. Available only in GastroPlus™ (Simulations Plus).	Open-source, implemented in GNU MCSim + R. Can be coded in any programming language.
Focus	Drug absorption with formulation-specific processes (dissolution, precipitation, transit, permeability, metabolism).	Environmental chemical absorption (assumes dissolved form; dissolution/precipitation steps removed).
Data inputs	Extensive drug-specific data (solubility, pKa, particle size, formulation, permeability, transporter activity).	Mainly *in-vitro* Caco-2 permeability (P_app_) with probabilistic scaling to *in-vivo* P_eff_ and gut-segment variability.
Calculation for C_ss_ *	$\frac{Dose}{GFR*f_{uBlood}+\frac{Q_{Liver}*f_{uBlood}*{CL}_{\mathrm{int}}}{Q_{Liver}+f_{uBlood}*{CL}_{\mathrm{int}}}}$	$\frac{Dose*F_{abs}*\left(1-E\right)}{GFR*\;f_{uBlood}+Q_{Liver}*E}$ , where E = $\frac{f_{uBlood}*{CL}_{\mathrm{int}}}{Q_{Liver}+f_{uBlood}*{CL}_{\mathrm{int}}}$
Output	Point estimates of fraction absorbed, oral bioavailability, systemic exposure.	Probabilistic distributions of Fabs and steady-state blood concentration (C_ss_) with confidence intervals.
Strengths	Well-established in regulatory science and pharmaceutical development, integrates detailed drug processes.	Flexible, transparent, open-source, tailored for chemicals lacking formulation data; explicitly handles uncertainty and variability.
Limitations	Not easily adaptable to chemicals outside pharma space, assumes complete absorption for many chemicals.	Current calibration relies heavily on drug datasets (limited environmental chemical data), assumes only passive absorption.

* C_ss_ = steady state concentration, Fabs = absolute bioavailability, and E = extraction ratio for first-pass metabolism into the term for the oral dose rate. Q_Liver_ = blood flow to liver, f_uBlood_ = unbound fraction of chemical in blood and CL_int_ , hepatic intrinsic clearance rate for first-order metabolism.

#### 2.3.2 Genome scale metabolic model integration with PBPK

Genome-scale metabolic (GSM) network models offer a comprehensive, mechanistic framework to represent and analyze the interactions between metabolites, enzymes, and transporters on a systems-wide scale (Lim et al., 2023). It includes all known metabolic reactions, genes and metabolites in an organism. The genome of the member species encodes the enzymes which catalyses different metabolic reactions. Since there are numerous microbiota community present inside human gut, the consideration of few whose genome are responsible for encoding enzymatic genes which are responsible for production of the metabolites of interest becomes very useful. To execute this, a community-based design algorithm (CoMiDA) was developed by Eng and Borenstein which designed simple microbial communities with predefined metabolic capabilities (Eng and Borenstein, 2016). Since metabolic pathways and rate of metabolism is a crucial information to be included while the development of a PBPK model, and due to alteration in these strains the mapping of these metabolic fluxes becomes important. There are substantial evidences that exposure of pollutants like PM10 associated with polycyclic aromatic hydrocarbons decreases the population of Firmicutes from 87.99% to 77.20% and increases the Bacteroidetes from 10.26% to 21.62% (Jeong et al., 2025). These strains along with Proteobacteria, Verrucomicrobia, and Actinobacteria are responsible for metabolism and digestion of most of the compounds entering out GIT (Yang K. et al., 2025). Through GSM modelling, the changes due to microbial metabolic interaction with the host can be studied.

This modeling approach has yielded successful studies; however, realizing personalized medicine and accurately predicting exposure scenarios for different chemicals will require continued methodological advances. In the previous section we discussed about the compartmentalization absorption and transit models which has the possibility to include the metabolic activity of predominant presence of those bacterial strains. In the same line, the ordinary differential equation based ACAT model along with GSM network models has proved its utility in estimating the spatiotemporal relationship between amino acids received from diet and absorption of levodopa in case of Parkinson’s disease (Guebila and Thiele, 2016).

Constraint based reconstruction and analysis (COBRA) is a framework that is used to enhance the comprehension of the large pool of meta-omics data generated from gut microbiome in a genome scale metabolic model. This can be used to mechanistically predict the metabolic fluxes caused by the microbiome in the gut (Heinken et al., 2021). There is an emerging application of this model in identifying the metabolism caused by microbial population acting as pathogens in human body and also to evaluate the antimicrobial treatment effect through metabolic shifts quantification which is done by calculating the reaction flux in a pathogen or the host cell (Zhu et al., 2022). The equations used in calculating the reaction flux is as follow (Equation 10):

$$S*V=0,a_{i}\leq v_{i}\leq b_{i},i=1,2,\ldots ,n$$ (10)

The same COBRA framework applied through COBRA toolbox was used to evaluate the drug-induced injury (isoniazid) considering whole-body drug exposure and the resulting biochemical responses at the cellular level (Cordes et al., 2018). Despite there being arguments about the advantage of GSM modelling. Quinn-Bohmann et al. (2025) suggests few limitations of these models. Single-organism or small-consortium GSMs are now routine, but scaling metabolic modeling to complex communities like the human gut remains difficult both computationally and in terms of experimental validation. Community-dynamic or multi-objective models can improve predictions over simple steady-state approaches, but they incur substantially higher computational cost, so model complexity should be chosen to match available data and use-case (Quinn-Bohmann et al., 2025).

### 2.4 Inter-species differences for microbial parameters

Introducing inter-species differences in microbiota based PBPK models are very important for evaluating alterations in compound metabolism and toxicity. Multiple factors like differences in gastrointestinal physiology, microbiota composition, and enzyme-activity can cause variation in outputs. The most prominent factor is the genetic variability between rodents and humans which strongly influences the microbiota environment present in the gut (Wos-Oxley et al., 2012). Phylum-level analysis reveals that humans and non-human primates exhibit a higher Firmicutes–Bacteroidetes ratio compared to mice and rats. Within these phyla, humans, shows abundance of the genus *Bacteroides* (phylum Bacteroidetes) are the most abundant, followed by Clostridiales (phylum Firmicutes) and family Ruminococcaceae. In contrast, the mouse gut is dominated by the S24-7 family (phylum Bacteroidetes) and Clostridiales (phylum Firmicutes), while rats and non-human primates show a greater presence of Prevotella (phylum Bacteroidetes) than mice and humans. Additionally, mice and rats have higher fecal lactate levels, whereas acetate is most abundant in human feces. These species-specific microbiota patterns across commonly used animal models highlight potential differences driven by host factors such as diet, genetics, sex, and age. The variation in the lifestyle and diet in humans contributes to high inter-individual variability unlike animal experiments that run in controlled environment and diet. This can lead to variability during model translation from animals to humans in real scenario and highlights the need for future studies that control these variables to better understand gut microbiome characteristics in preclinical models relative to humans (Nagpal et al., 2018). However, experimental evidence proves the microbiota environment of rat’s gut possess more similarity to humans compared to mice. This provides the base for choosing germ free rats for studying and translating animal model to humans for microbiota interaction studies (Flemer et al., 2017).

### 2.5 Advancements in capturing interindividual variability

In recent times, multiple advancements have been made to rigorously capture the interindividual variability, population PBPK frameworks that integrate virtual twin approaches offer a promising path toward individualized predictions. The shift from population-based to individual PBPK modelling, where individuals are modelled as virtual twins incorporating patient-specific physiological and microbial parameters, represents a key advancement toward precision dosing and clinical translation (Gonçalves et al., 2026). Such frameworks allow propagation of uncertainty in microbial parameters, such as Vmax and Km distributions, through population-level simulations, enabling derivation of credible intervals around predicted plasma concentration profiles, which is particularly valuable when microbial composition is a key source of pharmacokinetic variability.

Capturing the full scope of interindividual variability in microbial composition and activity within a population remains a key challenge for PBPK model development. Machine learning approaches are increasingly being explored to address this, offering the ability to predict candidate microbial activity and bacterial species involved in xenobiotic biotransformation potential from compositional or genomic data at scale. For instance, integrating chemoinformatics methods such as molecular descriptors (e.g., molecular weight, charge, valency), structural fingerprints, and structural similarity metrics (e.g., the Tanimoto coefficient, used to identify substrates structurally similar to an enzyme’s primary substrate) and machine learning methods enables the prediction of metabolic enzymes and bacterial species responsible for xenobiotic biotransformation (Malwe and Sharma, 2023).

Organ-on-a-chip microphysiological systems (MPS), which use human-derived cells within microfluidic devices to replicate physiologically relevant tissue environments, are increasingly coupled with AI for high-throughput, standardized 3D image analysis; for example, AI-driven pipelines have been used to reconstruct and quantify vascular network architecture and to track cell movement within organ-on-chip platforms via generative adversarial networks (GANs) (Lee et al., 2025). To date, this AI-MPS integration has been demonstrated for evaluating nanoparticle transport, toxicity, and biodistribution across several organ-specific chip platforms, rather than for gut-microbiome-specific applications. A similar concept could plausibly be adapted to a gut-microbiome-relevant chip to infer microbial kinetic parameters, though this specific application has not yet been demonstrated. Computational frameworks combining rule-based workflows and machine learning have demonstrated the ability to predict gut microbiota-mediated drug metabolism reactions by mapping compound structures to microbial enzyme databases (Chum et al., 2025). Furthermore, ML-PBPK hybrid platforms that integrate machine learning-predicted ADME parameters with physiologically based models have shown high predictive accuracy for human pharmacokinetics without requiring extensive *in-vitro* data (Li Y. et al., 2024). These predictive tools, however, depend on the size and composition of the training datasets used to build them; current databases of gut-microbial enzymes and xenobiotic-transformation reactions remain sparse and are skewed toward a small number of well-characterized bacterial taxa and druglike structures, which limits how confidently these predictions can be generalized to novel compounds or less-studied members of the microbiome. Graph neural network approaches such as SIMBA-GNN, which integrate mechanistic metabolic simulations with deep learning to predict microbial community composition and metabolite cross-feeding potential, represent another promising avenue for coupling microbiome dynamics with PBPK frameworks in a scalable and mechanistic manner (Aminian-Dehkordi et al., 2025). As with most predictive computational tools, the reliability of these models is largely confined to the chemical and microbial space represented in their training data; extrapolating predictions to structurally novel compounds or under-characterized bacterial taxa falls outside this applicability domain and introduces uncertainty that current microbiota-PBPK-ML applications do not yet systematically quantify.

### 2.6 Microbiota-specific model considerations, outputs, and validation

The PBPK model developed with the consideration of gut microbiota should be able to differentiate between the metabolites formed by microbiome and by intestinal and hepatic metabolizing enzymes. This can be done by estimating their C_max_ and hence their activity *in-vivo* in the form of therapeutic or toxic effect. It can result in differentiating microbiota-mediated metabolism, enabling more accurate and personalized PBPK predictions.

Some of the considerations for inclusion of microbiota in PBPK model are incorporation of compartments which hosts most of the microbiome and considerations of conversion coefficient of the parent compound into metabolites, and inclusion of elimination rate constant for both metabolite and the parent compound. The consideration of formation of secondary metabolite after primary metabolism by the gut microbiota and further compartmentalization of the tissues responsible for microbial enzymatic activity on the parent compound can help in improved pharmacokinetic prediction of compound. Building a sub-model for the major metabolite also strengthens but at the same time increases complexity in the model. Inclusion of factors like degradation half-life of the parent compound to estimate the intrinsic clearance as an input parameter is also one approach which can be applied based on the use case scenario (Kostantini et al., 2022; Mendez-Catala et al., 2020; Wang Q. et al., 2022; Zimmermann et al., 2019).

All these considerations clarify the role of metabolites in the bioactivity. For instance, incorporating microbial metabolism enabled the prediction of host plasma concentrations of S-equol and its conjugates. However, despite S-equol’s greater estrogenic potency, its overall contribution was found to be limited due to its significantly lower systemic levels compared to daidzein. Similarly, as a model output, Mendez-Catala et al. (2021) clarified that the predicted C_max_ values for α- zearalenone are less than 0.1% in rats and around 3% in humans relative to zearalenone. Despite α- zearalenone’s reported 60-fold greater estrogenic activity, its impact on *in-vivo* estrogenicity following zearalenone exposure is likely minimal due to its low systemic concentration. Simulation of special cases like ileostomy and/or colectomy on the metabolism of drugs like sulindac and sulfinpyrazone which are known for being metabolized by gut-microbiota was demonstrated by Kostantini et al. (2022). The model hence developed was to inform about the degradation half-life of the parent drugs which can be used to estimate the clearance in colon (Kostantini et al., 2022; Mendez-Catala et al., 2021; Wang Q. et al., 2022; Zimmermann-Kogadeeva et al., 2020).

Validation of microbiota-integrated PBPK models requires specific attention beyond standard pharmacokinetic model evaluation. Since microbial metabolism generates metabolites that may not arise from host metabolism alone, model performance must be assessed for both the parent compound and microbiota-derived metabolites simultaneously, using metrics such as predicted/observed Cmax ratios and cumulative urinary excretion ratios. For the ZEN PBK model, predicted urinary excretion ratios of 0.56 and 2.08 were obtained for a dose of 100 and 0.01 mg dose respectively (Mendez-Catala et al., 2021). While the daidzein/S-equol model achieved a 1.62-fold average difference for daidzein Cmax and a predicted/observed S-equol urinary excretion ratio of 0.89 at a dose ranging from 0.09 to 3.34 mg/kg bw daidzein (Wang Q. et al., 2022). Predictions within 0.5- to 2-fold of observed values are widely applied acceptance criterion for hepatic CYP-mediated PBPK models, including CYP3A-mediated drug-drug interactions (Yang C.-G. et al., 2025) and hepatic impairment models (Ladumor et al., 2023), though this has been described as a conventional limit applied in the absence of formally recommended guidelines rather than a criterion derived for a specific mechanism, with acknowledged limitations even within its original context. Notably, both the microbiota-informed PBPK model studies report these predicted/observed values with an explanation of differences to donor-to-donor variability and heterogeneity between literature studies, rather than validating them against a fixed pass/fail threshold (Mendez-Catala et al., 2021; Wang Q. et al., 2022). This indicates that a fixed fold-based criterion for simulated parameters is not yet established practice for microbiota-informed PBK models, and that the ratios reported above for microbiota-derived metabolites are better interpreted in this same descriptive, case-by-case manner than validated against any threshold of conventional acceptance criteria. Clinical scenarios involving surgical alteration of the GIT, such as ileostomy or colectomy, provide a particularly powerful validation tool, as they selectively eliminate colonic microbial metabolism, allowing direct attribution of systemic exposure differences to microbial biotransformation (Kostantini et al., 2022). Sensitivity analysis should specifically evaluate microbial kinetic parameters (Vmax, Km) and microbial biomass scaling factors, as these have been shown to exert a disproportionate influence on model predictions for microbiota-derived metabolites compared with host physiological parameters. For example, the large intestine microbial Vmax (VmaxLIEQUc) and fecal fraction of body weight (VMB) were identified as the dominant sensitive parameters for S-equol prediction, distinct from the hepatic parameters that dominated daidzein predictions (Wang Q. et al., 2022).

### 2.7 Proposed NAM framework

Drawing on the strategies reviewed in the preceding sections, we propose a structured NAM framework for integrating gut microbiota into PBPK models (Figure 2). This framework provides a practical, step-by-step pathway applicable to both drugs and environmental chemicals, reducing reliance on animal data while improving the accuracy of kinetic predictions.

**FIGURE 2:**
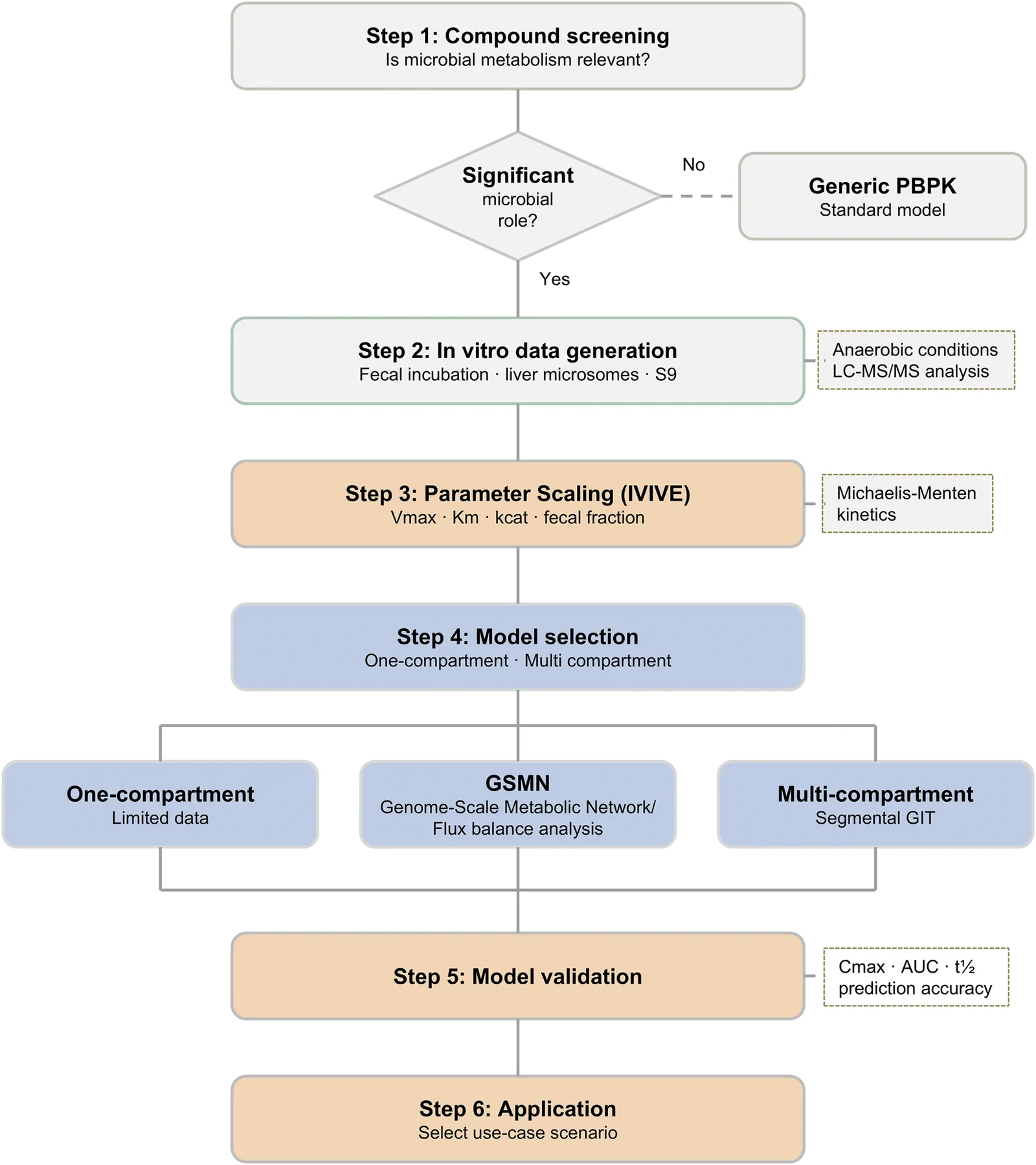
Proposed new approach methodology (NAM) framework for integrating gut microbiota into physiologically based pharmacokinetic (PBPK) models for drugs and environmental chemicals.

The first step involves screening of compounds to determine whether microbial metabolism plays a significant role in the overall ADME profile of the compound of interest. Such screening is needed because, although at least 50 drugs are already confirmed to be metabolized by gut bacteria, the true number is likely much higher, as no large-scale systematic screen for bacterial drug metabolism has yet been performed (Bisanz et al., 2018). Practical screening approaches include: (i) identification of the presence of functional groups, which are linked to microbial biotransformation, such as azo bonds, nitro groups, N-oxides, sulfoxides, and glycosidic or glucuronide/sulfate conjugates (Table 1) (Bisanz et al., 2018); (ii) direct, low-resource experimental screens, including *ex-vivo* anaerobic fecal incubation, culture-collection screening against representative gut bacterial strain panels, or fecalase (cell-free fecal enzyme extract) assays, which can detect substrate depletion or metabolite formation without requiring full kinetic characterization (Bisanz et al., 2018); (iii) pharmacokinetic signals from existing human or animal data, such as a secondary plasma concentration peak suggestive of enterohepatic recirculation, low oral bioavailability with a high fraction of unabsorbed dose reaching the colon, or an unexplained gap between total observed and characterized hepatic/renal clearance; and (iv) precedent from structurally or therapeutically related compounds already established as gut-microbiota substrates (Table 1). Compounds flagged by this screening as having microbial metabolism that meaningfully contributes to biotransformation, enterohepatic recirculation, or bioavailability should proceed through the full framework. When the microbial relevance is established, the second step involves *in-vitro* data generation using fecal incubation under anaerobic conditions, alongside hepatic metabolism assays using liver microsomes and S9 fractions. This provides an experimental basis for kinetic parameter estimation. In the third step, these *in-vitro* data are translated to *in-vivo* conditions through IVIVE and parameter scaling, applying Michaelis-Menten kinetics to derive Vmax, Km, and catalytic efficiency values scaled using species-specific fecal and tissue fractions. The fourth step involves the selection of an appropriate model structure based on data availability and study objectives. Options range from simplified one-compartment approaches to multi-compartmental absorption models such as ACAT or PECAT, or further integration with GSM network models for mechanistic pathway mapping. For complex microbial communities, the COBRA framework can be applied through the COBRA toolbox to predict metabolic fluxes driven by the microbiome, enabling mechanistic simulation of drug-induced metabolic perturbations and antimicrobial treatment effects at the cellular level (Equation 10). Following model development, the fifth step encompasses model validation through sensitivity analysis, uncertainty quantification, and comparison against clinical or *in-vivo* pharmacokinetic data, including key parameters such as Cmax, AUC, and t½. Finally, the sixth step defines the application scenario. Throughout all steps, regulatory reporting requirements from the USFDA, EMA, and OECD guidelines should be considered to ensure model qualification and acceptance. However, lack of standardized template of regulatory reporting requirement of microbiota assisted PBPK modelling is a roadblock in this regard. This has been discussed in more detail in Section 4.

## 3 Microbiota-based PBPK model applications

Microbiota-based PBPK model are emerging approach with immense application in predicting toxicity of environmental chemicals, personalized risk assessment, and chemical risk assessment (Table 3) (Zimmermann-Kogadeeva et al., 2020).

**TABLE 3:** Microbiota-based PBPK model applications; a guide to choose use case scenario with the required input parameters and desired outputs.

Application area	Modelling parameters	Inputs	Use cases	Outputs
Personalized dosing (sex/age/disease stratification; microbiome-aware)	GI transit and pH, gut microbial clearance/formation terms, hepatic/renal CL, protein binding, body-fat %, disease scalars	Sex and age-specific physiology, enzyme/transporter activity, albumin, microbiome composition/kinetics	Levodopa; dosing optimization in Parkinson’s disease patients. Irinotean dose adjustment based on individual’s glucuronidase activity.	Plasma/tissue C(t), AUC, C_max, age/sex differences, inter-individual variability, in drug exposure and toxicity risk.
Translational modeling (omics, kinetics, reverse translation)	Gut “metabolic” compartment; microbially driven k_cat/K_m; host inflammation scalars; metabolite formation	*Ex-vivo*/*in-vitro* microbial kinetics, relative abundances, host hepatic activity, inflammation markers	Microbiome-driven bioconversions integrated into PBPK frameworks	Predicted systemic metabolite levels; scenario analyses (antibiotics, diet shifts)
Compound–microbiota interactions (inactivation/activation; parent, metabolite)	Parallel gut microbial and hepatic pathways; enterohepatic circulation; permeability/partitioning updates	Microbial enzyme kinetics (azoreductase, decarboxylase, dehydroxylase, etc.), bile flow, f_u_	Levodopa, Digoxin, Irinotecan (parent and microbially formed metabolites)	Parent vs. metabolite exposure profiles; sensitivity of outcomes to microbiome variability
Environmental chemical risk (mixture pathways; chronic exposure)	Lifespan physiology; renal resorption; protein binding; slow exchange compartments	Cohort biomonitoring, diet/dust/water intakes, protein binding, renal transport	PFOS dynamic age-dependent PBPK (pediatric, adult, geriatric)	Dose reconstruction, organ burdens over decades, sensitive-group identification
NAMs and IVIVE (reduce animal use, link AOPs)	*In-vitro in-vivo* scaling of microbial and host kinetics; target-organ sub-compartments	*In-vitro* binding/partitioning, plastic/lipid binding corrections, cell-media conditions	Building qAOPs with TK underpinnings, organ-specific targets	Human-relevant equivalent concentrations at MIE sites; uncertainty bands

### 3.1 Personalized care

PBPK models enable the optimization of drug administration by ensuring that the drug is delivered at the right dose, for the appropriate disease, and at the required duration. By integrating patient-specific physiological and anatomical parameters, these models are consolidated as a key tool in the advancement of personalized medicine, contributing to minimize therapeutic failures and significantly reduce the occurrence of adverse effects (Hartmanshenn et al., 2016; Kuepfer et al., 2016). The development of personalized medicine can account for patients’ variability from the beginning. One interesting approach is integrating microbiota metabolism into PBPK models. As research has shown, the microbiota plays a critical role in the biotransformation of a wide range of compounds. However, its functionality and composition depend on many factors, including genetic polymorphism, age, sex, diet, health status and lifestyle. By integrating all these parameters into PBPK models, we can better predict individual responses and improve therapeutic strategies (Hartmanshenn et al., 2016). It should be noted that most personalization achieved to date relies on standard physiological and anatomical parameters rather than on microbiota-specific inputs. Microbiota-informed PBPK models have so far been demonstrated only as proof-of-concept for individual, well-characterized compounds such as zearalenone and daidzein (discussed in Section 2), and these still rely on population-averaged fecal incubation data rather than an individual’s own microbiome composition. True personalization at the level of a patient’s specific microbiome therefore remains to be achieved and will depend on resolving the IVIVE limitations discussed in Section 2.2.

Historically, male sex has often been prioritized in scientific studies, but there is growing interest in understanding the differences between males and females. It is well-establish that men and women exhibit distinct physiological responses, which increase the presence of adverse effects in women. This differences in adverse effects may be due to differences in body weight, gastrointestinal pH, transit and volume, affecting microbiota composition. In addition, women are in constant hormonal changes and tend to have higher body fat content, which modify the activity of hepatic enzymes and favours the accumulation of lipophilic drugs and, consequently, may require lower doses to observe therapeutic effects (Soldin and Mattison, 2009). While sex-based physiological differences are already routinely incorporated into standard PBPK models, the combination of sex-specific microbiota composition with PBPK modelling has not yet been demonstrated in practice and remains an emerging aspect of this application.

The parameter mentioned above are well-defined and well-characterized to capture individual differences that are essential for advancing in personalized medicine and PBPK models. However, all of them converge in alterations in the intestinal tract that would be affecting the bioavailability of drugs. An emerging interaction that required further attention is the drug-microbiota-diet axis, which plays a crucial role in influencing drug response and efficacy (Thiele et al., 2017). As reported above, diet is one of the most important factors influencing the state of microbiota. Consumption of artificial sweeteners, food additive or restrictive diets have been associated with an increase in *Bacteroides* and *Clostridia genera*, intestinal inflammatory processes or changes in metabolites produced by microbiota, while Mediterranean diet promote the growth of beneficial bacteria as well as greater diversity of micro-organism (Abou-Donia et al., 2008; Abrignani et al., 2024; Bian et al., 2017; Chassaing et al., 2015; Wu et al., 2016). In addition, this axis also contemplates drugs as a microbiota composition modulation. There is a wide variety of drugs that can evidence these changes, but one that is in the focus are antibiotics. It is well-known that antibiotics significantly affects microbiota balance and that the administrations of such compounds during pregnancy or early stages of life may have long-term effects in the microbiota (Lathakumari et al., 2024). At present, this axis is supported mainly by observational associations between diet, drugs and microbiota composition rather than by a quantitative model. Hence, PBPK model with integrated diet, drug and microbiota effects together, is a practically achievable goal which can be used to improve the accuracy of the model outputs.

Integrating this complex interplay into PBPK modelling promises to enhance preventive healthcare management by tailoring treatment not only to patient physiology but also to the dynamic influence of diet and microbiota on drug metabolism and efficacy. The approach could improve therapeutic outcomes and reduce adverse effects across divers patient populations. Standardized approaches to IVIVE that capture interindividual microbial variability rather than population averages, together with regulatory guidance for microbiota-informed PBPK models will be very helpful in designing the personalized dosing regimen for sensitive patients.

### 3.2 Translational modelling

In the last decades, research on microbiota has been growing generating large volumes of genomic, transcriptomic, proteomic and metabolomic data, to capture everything from the genetic composition of microbial communities to their metabolic functions and host interaction mechanism (Yang K. et al., 2025). The complexity and diversity of this data require advanced computational tools for the interpretation and application in clinical and pharmacological contexts. Among these, PBPK modelling along with GSM models can help in translating experimental data to real-case scenarios. For instance, Zimmermann et al. (2019) used conventional and germ-free mice to study clonazepam, an anxiolytic and anticonvulsant drug with a complex metabolic pattern (Zimmermann et al., 2019). This drug is reduced in the gut, producing aminoclonazepam and aminohydroxyclonazepam. This data allowed the generation of a host-microbial pharmacokinetic model that predicted a significant microbial contribution of about 70% in the production of both metabolites. In addition, they employed PBPK models to assess how inflammation alters the pharmacokinetics of irinotecan and its active metabolite SN-38 (Tao et al., 2022). These models can be further enriched by integrating omics-derived data, such as microbial abundance, tissue-specific expression or host hepatic activity. This allows for simulation of complex scenarios, including microbiota induced by antibiotics, diet or specifics host characteristics.

Furthermore, reverse-translation approaches, where clinical or *in-vivo* pharmacokinetic and toxicity data feedback, refine *in-vitro*, *in silico* and preclinical models, represent a key opportunity for risk assessment and mechanism understanding. This allows earlier identification of drug interactions or microbiome-mediated toxicity, dose optimization as well as revealing new therapeutic targets.

### 3.3 Compound-microbiota interactions

Microorganism presents in the gut microbiota possess a wide diversity of enzymes that allow them to modify the structure of chemicals such as drugs and environmental pollutants prior to their absorption in the GIT (Chen et al., 2022). One example of microbiota metabolism and drug breakdown is Digoxin, prescribed for heart conditions like atrial fibrillation and arrythmias. This drug is inactivated by the action of actinobacteria *Eggerthella lenta* in the gut. Specifically, digoxin is reduced to its inactive metabolite dihydrodigoxin, which loses its ability to bind to the Na+/K + ATPase pocket, diminishing its therapeutic efficacy (Haiser et al., 2013; Haiser et al., 2014). Similarly, another well-known drug transformation is levodopa, which is used to treat Parkinson’s disease. This drug can be transported to the GIT or the central nervous system (CNS), but more than half of Levodopa does not reach the brain, because bacteria with the tyrosine decarboxylation gene are able to decarboxylate Levodopa to dopamine and then, the bacterium strain *E. lenta* dehydroxylate dopamine into *m*-tyramine, reducing their bioavailability (Zhong et al., 2023; Menozzi and Schapira, 2024). In addition, there are other compounds like metformin that have a bidirectional drug-microbiota interaction. This is because the effectiveness of metformin depends on the basal microbial profile of the host, but the drug also modulates the composition by increasing *Akkermansia muciniphila* strain (Cuesta-Zuluaga et al., 2017; Wu et al., 2017). All these drugs can be modeled using PBPK model to understand the influence of pharmacokinetics on the efficacy considering microbiome interactions.

Environmental chemicals with daily exposure are also biotransformed by microbiota. Here we find compounds such as polychlorinated biphenyls (PCBs) which are implicated in enterohepatic recirculation and were associated with intestinal dysbiosis, which modulates their toxicity (Rude et al., 2019). Another toxic substance to which humans are constantly exposed by food ingestion is pesticides. There are a wide variety of pesticides, but the most commonly used are organophosphate, which causes changes in bacterial population and composition, leading to inflammation and alterations in the energy metabolism in the gut (Ali and AlHussaini, 2024). Microbiota-based PBPK models can be applied to evaluate compound-microbiota interactions and understand the toxicokinetic profile.

On the other hand, pro-toxics and pro-pharmacological compounds can be activated by the gut microbiota. Activation of these compounds either enhances their therapeutic effect or increases their risk of adverse reactions. Hence, consideration of microbiota already provides pharmaceutical developers to design molecules which become active after their interaction with microbiota. A well-known example of microbiota-dependent drug activation is sulfasalazine. This prodrug is commonly used in the treatment of inflammatory bowels and rheumatoid arthritis. When sulfasalazine reaches the gut, it is reduced by bacterial azoreductases, particularly those of genera *Clostridium* and *Bacteroides*. The reduction reaction produces two active metabolites: sulphapyridine and 5-aminosalicylic acid, the latter triggering the therapeutic effect (Sousa et al., 2008). Similarly, lactose keto analogue, lactulose, is hydrolyzed by intestinal bacteria, producing lactic acid and acetic acid. Both acids lower the pH of the gut protonating ammonia and other amines, which reduces their absorption and increases their excretion in the feces (Sousa et al., 2008).

Therefore, it is important to include the interactions of the microbiota with the different chemical compounds in PBPK models as it allows us to predict more accurately the effect of these compounds on the organism and thus promote safer and more personalized therapeutic strategies.

### 3.4 Compound-microbiota-diet axis

This section addresses a distinct and PBPK-relevant aspect of the diet-microbiota relationship. Diet functions both as a class of compounds subject to the same microbial biotransformation mechanisms described above (Section 3.3), and, more importantly, as a constant exposure that continuously shapes the microbial composition and enzymatic capacity available to metabolize co-administered drugs. This dual role makes diet a relevant covariate for microbiota-informed PBPK models, distinct from treating any single dietary compound as an isolated substrate. Food components and dietary supplements, similar to pharmaceuticals and environmental chemicals often require gut microbial biotransformation to facilitate their absorption and biological activity. (Qadri et al., 2024).

The conversion of soy isoflavone daidzein into equol is a clear example of this activation of the microbiota. The conversion of daidzein occurs through the action of bacteria belonging to Coriobacteriaceae family. When equol is obtained, it exerts its effects by improving cardiovascular health, reducing menopausal symptoms and providing bone protection, as well as preventing hormone-dependent cancers (Setchell and Carlo, 2010; Kobayashi et al., 2013).

Furthermore, there are bioactive compounds found in plant-based food, such as berries, nuts, and pomegranates, which require the action of the microbiota to be absorbed. This is the case with ellagitannins, which in their native form cannot be absorbed due to their large size and polarity. Therefore, the gut microbiota acts by biotransforming ellagitannins into urolithins in a two-step process. The active metabolite, urolithins, enters the enterohepatic circulation and exerts multiple health-protective effects due to its antioxidant, anti-inflammatory and anti-tumor properties (Banc et al., 2023).

However, different aspects need to be considered when considering the transformation of dietary compounds. It is very important to take into account that each person has a different gut microbiota composition, which will affect the production and bioavailability of both equol and urolithins, as well as other compounds. This microbial composition is affected by various factors such as age, sex, weight, diet, physical activity and health status of the individual (Banc et al., 2023; Setchell and Carlo, 2010).

## 4 Regulatory prospective for microbiota integrated PBPK models

From a regulatory standpoint, incorporating microbial metabolism, particularly that mediated by the gut microbiota into PBPK models is becoming nontrivial due to its significant influence on the environmental persistence and internal exposure profiles of chemicals. Despite having enough evidence related to microbiota playing an important role in metabolism, no standalone guidance on microbiota-informed PBPK model exists. The OECD guidance document on the characterisation, validation and reporting of Physiologically Based Kinetic (PBK) models for regulatory purposes gives emphasis on the effect of microbiota on the biliary excretion of the chemicals which undergoes enterohepatic recirculation. This can be accounted for the chemicals where the metabolite is converted back into the parent chemical by the gut microbiota, leading to its reabsorption in the colon and hence the having a prolonged effect of the chemicals (OECD, 2021). The EMA provides the guidelines on the reporting PBPK modelling and simulations. Though it does not exclusively mentions about gut microbiota, there is discussion about fraction unbound in microsomes, which if deemed important for the model can be determined experimentally or predicted (European Medicine Agency, 2018). Also, the relationship between the liver microsomal fraction, metabolic enzymes and the effect of microbiota on the enzymatic activity is very well established (Jourová et al., 2020), hence it can be said that EMA indirectly considers the involvement of microbiota in the model development. The USFDA in its guidance to industry for PBPK analysis does not enforce any standard PBPK model reporting framework but emphasizes on the clear mention of how the conducted PBPK analysis answers the specific scientific question in a clinical setting (USFDA and CDER, 2018). But as such clear reporting guidelines or acceptance criteria for microbiota-based PBPK model is lacking which can guide developers towards developing robust and harmonized models. Key aspects which need to be included in such modules are microbial metabolic kinetic inputs, especially standardized way of performing IVIVE of such parameters, scaling microbial biomass, and microbial clearance in different gut compartments, output validation and sensitivity analysis. For appropriate prediction of impact of exposure on the host, it is important to standardize the microbial metabolism, including the parameterization of PBPK model.

With the emergence of NAM, incorporation of gut-on-chip and liver-on-chip micro physiological system data can really help in moving towards *in-vitro* supported microbiota PBPK models. Arguments have been made consistently in favor of upgrading the reporting template of PBPK models by incorporating environmental chemicals relevant elements. Regulators need to include these approaches in guidelines to be developed for standalone microbiota PBPK Models. One good approach can be identifying the compounds where microbiota plays an important role and classifying them into different tiers based not only on percentage of microbial metabolism but also on the pharmacological or toxicological significance of the resulting metabolite(s). Toxicological significance of metabolism is important because even a small fraction of microbial conversion can be clinically important if it generates a highly active or toxic metabolite. This is illustrated by irinotecan, where gut microbial β-glucuronidase-mediated deconjugation regenerates the potent metabolite SN-38 locally within the intestinal lumen, causing severe gastrointestinal toxicity despite representing only a fraction of the drug’s overall clearance (Table 4). Conversely, zearalenone and daidzein form more potent microbial metabolites (α-zearalenol and S-equol, respectively), but these remain below concentrations expected to cause clinically relevant effects because of their low systemic levels (Section 2.6). For the success of such strategies, it should be obligatory for the manufacturer to submit microbial metabolism information in the dossier. This will help in the classification of compounds. Gut microbiota, through its metabolizing capacity affects the chemical which is summarized in Table 4.

**TABLE 4:** Examples of drugs with substantially effected due to microbial metabolism.

Microbiome mediated activity	Examples	Effect	References
Prodrug activation	Sulfasalazine	5-aminosalicylic acid generated by azoreductase enzyme secreted due to *Lactobacillus* and *Bifidobacterium* strains present in the colon.	Crouwel et al. (2021)
	Lovastatin	Gut bacteria hydrolyze the lactone ring of lovastatin to form active hydroxy acid form, increasing the systemic active metabolite exposure. Antibiotics that disrupt gut microbiota, reduce this activation, demonstrating the microbiota-dependent contribution in prodrug activation.	Yoo et al. (2014)
Inactivation	Levodopa	Gut bacterial aromatic l-amino acid decarboxylase enzyme converts levodopa to dopamine in intestine, hence reducing the fraction of levodopa available for systemic absorption and CNS delivery. This peripheral decarboxylation decreases neuronal dopamine synthesis in brain.	Cheng et al. (2024)
Altered drug absorption	Amiodarone	Coadministration of *Escherichia coli N*issle 1917 strain with amiodarone is related to its enhanced bioavailability probably due to change in intestinal pH hence affecting its ionization.	Matuskova et al. (2014)
Deconjugation	Irinotecan	Hepatic conversion of irinotecan to inactive metabolite SN-38G, which is excreted into the gut. Gut microbial β-glucuronidases then deconjugate SN-38G, regenerating the active SN-38 within the intestinal lumen leading to gastrointestinal toxicity, particularly delayed diarrhea.	Wallace et al. (2010)

The consideration of microbial metabolism can play an even crucial role in the cases of drug-drug interaction. Regulatory expectations for model qualification in DDI assessments demand that PBPK platforms demonstrate predictive accuracy for both perpetrator and victim scenarios. This includes accurate differentiation of site-specific enzyme inhibition (e.g., hepatic vs. intestinal CYP3A4), prediction of key pharmacokinetic parameters (C_max_, AUC, t1/2), and confirmation of metabolic pathways using clinical or pharmacogenetic data (European Medicine Agency, 2018). However, such frameworks often overlook the role of the gut microbiota in modulating drug metabolism and clearance, especially for orally administered compounds undergoing EHR or microbial biotransformation. Given that microbial enzymes may contribute significantly to first-pass or systemic metabolism either by directly metabolizing xenobiotics or by regenerating parent compounds from conjugates, failure to incorporate these pathways may compromise the model’s completeness. For regulatory acceptance, microbiota-informed PBPK models must demonstrate that inclusion of gut microbial metabolism improves prediction quality for compounds where such contributions are mechanistically plausible. As regulatory agencies require PBPK models to be qualified for specific enzymes, anatomical sites, and populations, inclusion of microbiota-mediated processes introduces a parallel need for qualification and validation, particularly when microbial metabolism alters drug exposure in a manner comparable to hepatic or intestinal enzymes (Table 4).

## 5 Future directions

Microbiota-based PBPK modelling holds great promise for integrating host physiology with microbial metabolism to improve kinetic predictions. With increasingly sophisticated *in silico* models and availability of high-throughput *in-vitro* data, it is becoming feasible to incorporate real-time or scenario-based changes in microbial activity caused by diet, chemical exposure or drugs like antibiotics. It will also be important to incorporate bidirectional signalling or feedback loops into PBPK frameworks. For instance, short chain fatty acids (SCFAs), metabolite of gut microbiota have a bidirectional gut-liver relationship and can affect enzymatic function of liver and integrity of gut barrier by immune cell activity, tight cell junctions and controlling hypoxia-inducible factors (Li X. et al., 2024). These processes in turn can affect metabolism and excretion of xenobiotics, sometime leading to toxicity or increased bioavailability. Similarly, a close relationship exists between antibiotic efficacy and bacterial metabolism. Antibiotics can cause gut dysbiosis which in turn can alter metabolism of antibiotics, similarly metabolic state of bacteria affects the susceptibility to harmful effects of antibiotics and these effects differs from bactericidal and bacteriostatic antibiotics (Stokes et al., 2019). Modelling the pharmacokinetics depending on variable factors like type of antibiotics, duration of treatment and altered microbial species can provide interesting results to move towards personalized treatment. Realizing this bidirectional, feedback-inclusive modelling will, however, depend on generating dedicated kinetic and dose-response data for these microbiota-host feedback loops, which are largely lacking at present and currently limit how quantitatively these mechanisms can be incorporated into a working model.

Incorporating inter-individual variability especially different microbiota composition profile as well as host metabolic capacity, and gut transit time can help in building personalized and population-based models. Shotgun metagenomic sequencing can help in comparing gut microbiome composition and function between individuals which in turn can develop a robust database about population and further can be linked to microbiota PBPK models (Wang Y. et al., 2022). Availability of multi-omics data especially metatranscriptomics, metaproteomics and metabolomics can help in identifying altered RNA, protein and metabolites providing insight about alteration in biological pathway and disease (Muller et al., 2024). Some of this data can be coupled with microbiota PBPK models utilizing advanced toolkits for multi-omics like mixOmics, integrated meta-omic pipeline (IMP), MiBiomics which are helpful for connecting multi-dimensional data to identify microbiome-disease interactions (Arıkan and Muth, 2023). Realizing this potential will require multi-omics datasets that are standardized and drawn from sufficiently diverse populations. Most currently available gut-microbiome multi-omics datasets are limited in cohort size and diversity and lack harmonized processing pipelines, which constrains how representative any resulting population-level database would be at present.

## 6 Conclusion

PBPK models have been used for more than 5 decades to characterize the ADME behaviour of drugs and chemicals, but despite growing evidence that microbiota influences drug and chemical metabolism, microbiota-informed PBPK applications limited. Accumulating evidence shows that microbial metabolism can meaningfully alter systemic concentrations, bioactivation, detoxification, and enterohepatic recirculation, with direct implications for both pharmacology and toxicology. Incorporating these processes into PBPK models is therefore essential for improving predictions of efficacy, safety, and population variability. This review has outlined the parameters needed to build microbiota-informed PBPK models and highlighted uncertainties related to differences in microbial composition, gut physiology and host susceptibility. Looking forward, the integration of multi-omics data, GSM and COBRA-based microbiome models, bidirectional gut–liver signalling, and population PBPK frameworks, supported by machine learning for microbial parameter inference, will be key to capturing the full impact of the microbiota on xenobiotic kinetics. By assembling these methodological, computational, and regulatory perspectives, this review provides a practical roadmap for researchers and regulatory scientists seeking to develop and apply microbiota-based PBPK models as NAMs in support of personalized medicine and modern chemical risk assessment. In parallel, transitioning from generic to personalized PBPK models can improve prediction of toxicity and support individualized dosing regimens, while dedicated regulatory guidelines for microbiota-informed PBPK will needs to be refined and harmonised to facilitate wider regulatory acceptance.
